# Successful thoracoscopic operative approach for refractory pneumothorax in interstitial lung disease under local anaesthesia

**DOI:** 10.1002/rcr2.1331

**Published:** 2024-03-25

**Authors:** Shinya Yamanaka, Clara So, Naoki Nishimura, Tomoaki Nakamura, Shinsaku Kabemura, Ryosuke Kumagai, Kohei Okafuji, Atsushi Kitamura, Fumitsugu Kojima, Yutaka Tomishima, Torahiko Jinta, Toru Bando

**Affiliations:** ^1^ Department of Internal Medicine St. Luke's International Hospital Tokyo Japan; ^2^ Department of Pulmonary Medicine, Thoracic Center St. Luke's International Hospital Tokyo Japan; ^3^ Department of Thoracic Surgery, Thoracic Center St. Luke's International Hospital Tokyo Japan

**Keywords:** autologous blood patch, endobronchial watanabe spigot, interstitial lung disease, refractory secondary pneumothorax, video‐assisted thoracic surgery

## Abstract

Refractory pneumothorax associated with interstitial lung disease (ILD) remains a challenging condition due to the patient's tolerability and lung compliance that restrict the feasibility of aggressive interventions. Additionally, many cases recur after improvement with treatment, and reports of successful management for this complicated condition are limited. Herein, we report the case of a 60‐year‐old man with ILD, utilizing home oxygen therapy, who experienced a successful recovery from a surgical intervention under local anaesthesia for pneumothorax. This case highlights the potential for operative intervention under local anaesthesia as a viable option for patients who do not respond to internal approaches.

## Graphical abstract

Refractory pneumothorax associated with interstitial lung disease (ILD) remains a challenging condition due to the patient's tolerability and lung compliance that restrict the feasibility of aggressive interventions. Additionally, many cases recur after improvement with treatment, and reports of successful management for this complicated condition are limited. We report the case of a 60‐year‐old man with ILD, utilizing home oxygen therapy, who experienced a successful recovery from a surgical intervention under local anaesthesia for pneumothorax.

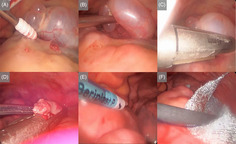

## INTRODUCTION

Pneumothorax occurs in 20% of patients with interstitial lung disease (ILD), and the prognosis is poor.^1^ Some therapeutic options for pneumothorax include autologous blood patch, pleurodesis, replacement of Endobronchial Watanabe Spigot (EWS), or surgery. However, a substantial recurrence rate of 30%–80% persists after initial treatment,^2^ complicating the management of pneumothorax with ILD. Surgery is a reasonable option for a patient who has continuous leakage, but the need for general anaesthesia is a major obstacle.

In this case, the use of local anaesthesia for thoracoscopic surgery is a viable and safe option. And, if we tend to do video‐assisted thoracoscopic cystectomy under local anaesthesia, identifying leakpoint is challenging due to limited vision. Herein, we report a case of refractory pneumothorax that was successfully treated under local anaesthesia with the aid of thoracoscopic contrast to identify the lesion and cure the patient.

## CASE REPORT

A 60‐year‐old man presented to our hospital with dyspnea. He had a 42‐pack‐year smoking history and was diagnosed with CPFE (combined pulmonary fibrosis and emphysema) and S6 squamous cell carcinoma (pT2aN1M0, p‐stage IIB) of the left lung. He underwent a left lower lobectomy and achieved a recurrence‐free period of 5 years. However, his dyspnea progressed slowly with CPFE, and he was started on nintedanib. Home oxygen therapy was introduced a year ago, and oral morphine was initiated 1 month before visiting our hospital.

**FIGURE 1:**
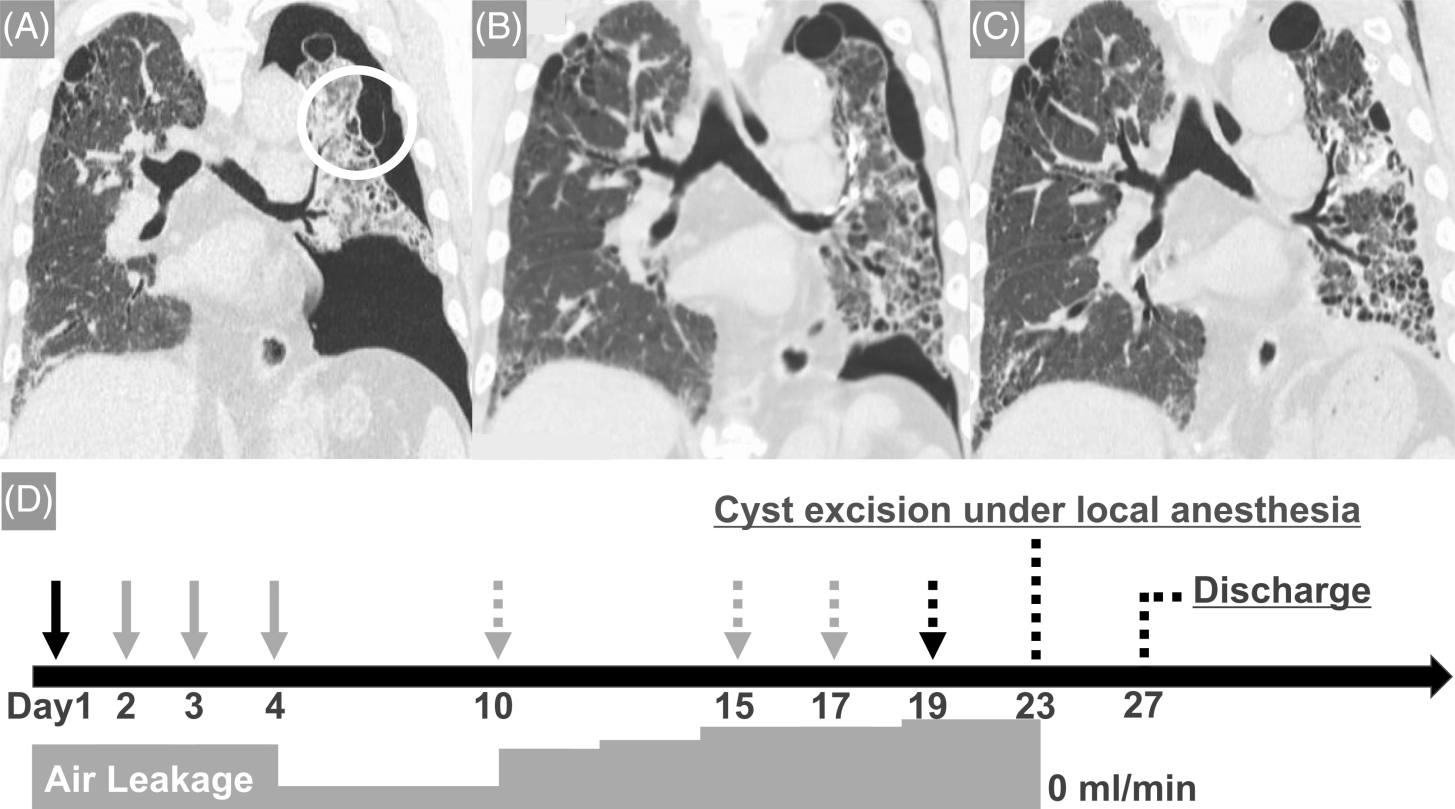
(A) On the day of admission, CT revealed the left pneumothorax. The white circle indicates a cyst with a suspected leak. (B) On day 17, CT revealed lungs without expansion after the replacement of Endobronchial Watanabe Spigot (EWS). (C) On day 23, CT revealed an expanded lung obtained after the operation. (D) A summary of the patient's clinical course. The black arrow: thoracic drain placement. The grey arrow: pleural blood patching. The grey dotted arrow: replacement of EWS into apicoposterior segment bronchus (B1/2). The black dotted arrow: left thoracography. CT, computed tomography.

When he arrived at our hospital, he exhibited tachypnea and desaturation. On physical examination, we observed shortened expiratory time, loss of breath sounds on the left side, and fine crackles in the lower lungs. Blood tests revealed elevated LDH and Krebs von den Lungen‐6 (Table 1). Radiographic examinations indicated a left pneumothorax with mediastinal variations, and leakage from a cyst in the left upper lobe was suspected (Figure 1A). Immediately, a drain was placed, and leakage was managed with a portable thoracic drainage device (Thopaz, Medela Healthcare). Daily autologous adhesion did not succeed. A total of three EWS replacements were performed to fill the left apicoposterior segment bronchus (B1 + 2), but no response was achieved (Figure 1B). To search for the responsible lesion, thoracography was performed and a leak was suspected in the mediastinal measurement of the upper lobe of the lung.

**TABLE 1:** Laboratory data on admission.

Variable	Reference range	This case
Blood		
Haemoglobin (g/dL)	11.6–14.8	16.2
White blood cell count (×10^3^ per μL)	3.3–8.6	10.6
Platelet count (×10^4^ per μL)	158–348	212
Total protein (g/dL)	6.6–8.1	7.0
Albumin (g/dL)	4.1–5.1	3.7
Total bilirubin (mg/dL)	0.4–1.5	1.2
Aspartate aminotransferase (U/L)	13–30	55
Alanine aminotransferase (U/L)	10–42	59
Lactate dehydrogenase (U/L)	124–222	304
Alkaline phosphatase (U/L)	38–113	66
Blood urea nitrogen (mg/dL)	8.0–22	11.9
Creatinine (mg/dL)	0.46–0.79	1.03
Sodium (mEq/L)	138–145	142
Potassium (mEq/L)	3.6–4.8	4.2
Chloride (mEq/L)	101–108	107
Sialylated carbohydrate antigen KL‐6 (U/mL)	<500	1106
C reactive protein (mg/dL)	<0.14	0.26

**FIGURE 2:**
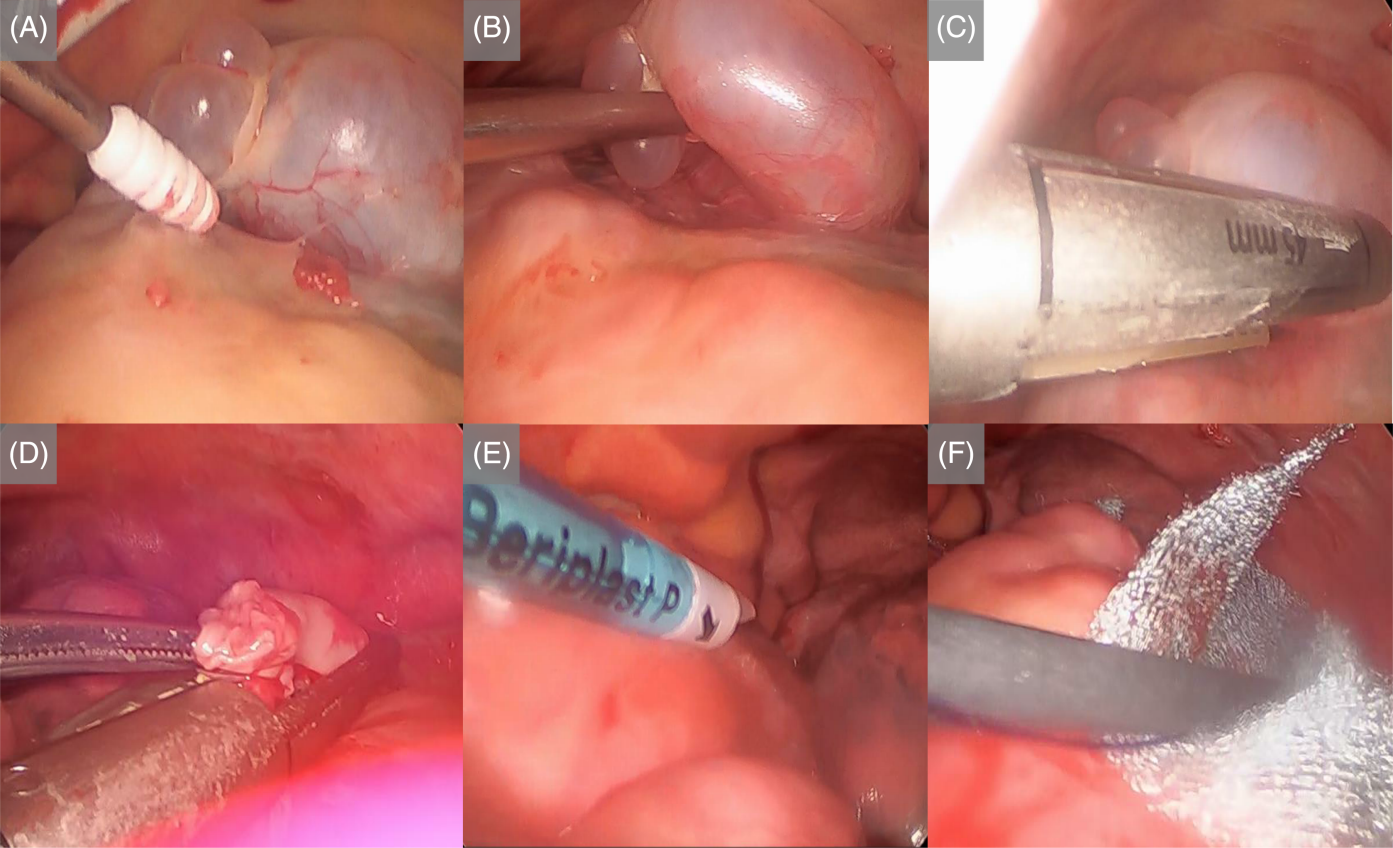
Operative record. (A, B) Observation of the thoracic cavity revealed multiple cysts, including a giant cyst on the outer side of the pulmonary apex. (C, D) The lateral giant cyst was resected, and a good field of view was obtained. (E) A fibrin glue (Beriplast(R)P, CSL Behring) was applied to the cyst and staple line on the mediastinal side of the pulmonary apex. (F) Polyglycolic acid sheet (Neoveil, Gunze medical) was applied to the same area.

A thoracoscopy under local anaesthesia was planned considering the lung condition. Anaesthesia was provided by shallow sedation with remimazolam, epidural catheter (Th 5/6), and local anaesthesia. The patient was positioned in the right lateral recumbent position with three trocars (on the anterior axillary line of the second intercostal space, on the midaxillary line of the fourth intercostal space, and in the para mammary region). Intraluminal observation revealed multiple cysts, including an outer giant cyst at the apex of the lung. The lateral giant cyst was excised, and a good visual field was obtained. However, no obvious leak was identified. A 20 French thoracic drain was placed in the direction of the left lung apex after applying A fibrin glue (Beriplast(R)P, CSL Behring) and Polyglycolic acid sheet (Neoveil, Gunze medical) to the cyst and a staple line on the mediastinal side of the lung apex, which was suspected as a lesion based on thoracoscopy. The procedure was terminated after confirming good lung expansion and proper drain position with bilateral lung ventilation. The operation time was 1 h.43 m., and the operative record is shown in Figure 2. At the end of the surgery, the leak volume was zero, and after confirming that the lungs were well dilated (Figure 1C), the chest drain was removed on the second postoperative day, and the patient was discharged on the fourth postoperative day. The clinical course of this case is shown in Figure 1D.

Following a period of normalcy post‐surgery, the patient encountered a recurrence 6 months later. Drawing from previous experience, we detected the lesion in the lower lobe of the left lung. And, the patient underwent a subsequent video‐assisted thoracoscopic cystectomy under local anaesthesia, that led to the immediate resolution of the pneumothorax. Thereafter, the patient remained free of recurrence for 6 months following the second surgical intervention.

## DISCUSSION

Surgical advancements, such as single‐lung ventilation and video‐assisted thoracic surgery (VATS), have significantly enhanced the prognosis of patients facing challenging medical conditions. However, these procedures present a notable challenge due to the necessity of general anaesthesia. During intubation of ILD patients, high positive pressure ventilation is required due to poor lung compliance. Prolonged positive pressure ventilation may lead to lung pressure damage and re‐emergence of air leak, and poor prognosis. In fact, surgery for pneumothorax in patients with background diseases such as ILD results in death in 4%–21% cases.^3^, ^4^

On the other hand, as a long‐term prognosis can be expected if the surgery is safely completed, VATS with local anaesthesia becomes a saviour. This procedure can deal with peripheral lung and pleural lesions, which are visible without requiring strong lung compression. However, when intending to perform a procedure with toponarcosis, several limitations arise due to the challenges associated with incomplete one‐lung ventilation: Difficulty in leak testing, constrained workspace, and the risk of cough reflection. Therefore, it is crucial to identify the lesion and enhance treatment certainty by thoracography beforehand.^5^

In our case, successful surgery was performed for a recurrent secondary pneumothorax. This indicates that surgery under local anaesthesia can be performed safely. Pulmonary physicians should be aware of the potential for operative intervention under local anaesthesia as a viable and attractive option for patients who do not respond to internal medicine approaches.

## AUTHOR CONTRIBUTIONS

*Conceptualization*: Clara So. *Investigation*: Shinya Yamanaka, Clara So, Naoki Nishimura, Tomoaki Nakamura, Shinsaku Kabemura, Ryosuke Kumagai, Kohei Okafuji, Atsushi Kitamura, Fumitsugu Kojima, Yutaka Tomishima, Torahiko Jinta, Toru Bando. *Writing—original draft*: Shinya Yamanaka, Clara So, Naoki Nishimura. *Writing—review and editing*: Shinya Yamanaka, Clara So, Naoki Nishimura, Fumitsugu Kojima, Toru Bando.

## CONFLICT OF INTEREST STATEMENT

None declared.

## ETHICS STATEMENT

The authors declare that appropriate written informed consent was obtained for the publication of this manuscript and accompanying images.

## Data Availability

The data that support the findings of this study are available from the corresponding author, SY, upon reasonable request.
